# Implementing and evaluating a regional strategy to improve testing rates in VA patients at risk for HIV, utilizing the QUERI process as a guiding framework: QUERI Series

**DOI:** 10.1186/1748-5908-3-16

**Published:** 2008-03-19

**Authors:** Matthew B Goetz, Candice Bowman, Tuyen Hoang, Henry Anaya, Teresa Osborn, Allen L Gifford, Steven M Asch

**Affiliations:** 1 Infectious Diseases Section (111-F), VA Greater Los Angeles Healthcare System, Los Angeles, California, USA; 2 VA San Diego Healthcare System, San Diego, California, USA; 3 General Medicine (111G), VA Greater Los Angeles Healthcare System, Los Angeles, California, USA; 4 Veterans Integrate Service Network 22, Long Beach, California, USA; 5 VA Bedford Center for Health Quality, Outcomes and Economic Research, Edith Nourse Rogers Memorial Veterans Hospital, Bedford, Massachusetts, USA

## Abstract

**Background:**

We describe how we used the framework of the U.S. Department of Veterans Affairs (VA) Quality Enhancement Research Initiative (QUERI) to develop a program to improve rates of diagnostic testing for the Human Immunodeficiency Virus (HIV). This venture was prompted by the observation by the CDC that 25% of HIV-infected patients do not know their diagnosis – a point of substantial importance to the VA, which is the largest provider of HIV care in the United States.

**Methods:**

Following the QUERI steps (or process), we evaluated: 1) whether undiagnosed HIV infection is a high-risk, high-volume clinical issue within the VA, 2) whether there are evidence-based recommendations for HIV testing, 3) whether there are gaps in the performance of VA HIV testing, and 4) the barriers and facilitators to improving current practice in the VA.

Based on our findings, we developed and initiated a QUERI step 4/phase 1 pilot project using the precepts of the Chronic Care Model. Our improvement strategy relies upon electronic clinical reminders to provide *decision support*; audit/feedback as a *clinical information system*, and appropriate changes in *delivery system design*. These activities are complemented by academic detailing and social marketing interventions to achieve *provider activation*.

**Results:**

Our preliminary formative evaluation indicates the need to ensure leadership and team buy-in, address facility-specific barriers, refine the reminder, and address factors that contribute to inter-clinic variances in HIV testing rates. Preliminary unadjusted data from the first seven months of our program show 3–5 fold increases in the proportion of at-risk patients who are offered HIV testing at the VA sites (stations) where the pilot project has been undertaken; no change was seen at control stations.

**Discussion:**

This project demonstrates the early success of the application of the QUERI process to the development of a program to improve HIV testing rates. Preliminary unadjusted results show that the coordinated use of audit/feedback, provider activation, and organizational change can increase HIV testing rates for at-risk patients. We are refining our program prior to extending our work to a small-scale, multi-site evaluation (QUERI step 4/phase 2). We also plan to evaluate the durability/sustainability of the intervention effect, the costs of HIV testing, and the number of newly identified HIV-infected patients. Ultimately, we will evaluate this program in other geographically dispersed stations (QUERI step 4/phases 3 and 4).

## Background

Over the past decade, in the developed world, the Human Immunodeficiency Virus (HIV) has been transformed from a fatal disease with rapid progression to clinical AIDS (Acquired Immune Deficiency Syndrome) and death – to a chronic illness that can be successfully managed through outpatient-based care over long periods of active life. However, advances in the effectiveness of treatment have been accompanied by new challenges for healthcare systems as they aim to provide adequate chronic illness care to large numbers of ambulatory HIV patients.

Unfortunately, the Centers for Disease Control and Prevention (CDC) estimates that approximately 25% of the nearly one million HIV-infected persons in the United States are not aware of their status [1]. Thus one of the key barriers to the receipt of care for HIV infection is under-diagnosis of this condition, particularly in the early, asymptomatic phases of illness when medical intervention is highly cost-effective [2,3]. This article is one in a *Series *of articles documenting implementation science frameworks and approaches developed by the U.S. Department of Veterans Affairs (VA) Quality Enhancement Research Initiative (QUERI). QUERI is briefly outlined in Table 1 and is described in more detail in previous publications [4,5]. The *QUERI Series' *introductory article [6] highlights aspects of QUERI related specifically to implementation science, and describes additional types of articles contained in the *Series*.

**Table 1:** The VA Quality Enhancement Research Initiative (QUERI)

The U.S. Department of Veterans Affairs' (VA) Quality Enhancement Research Initiative (QUERI) was launched in 1998. QUERI was designed to harness VA's health services research expertise and resources in an ongoing system-wide effort to improve the performance of the VA healthcare system and, thus, quality of care for veterans.

QUERI researchers collaborate with VA policy and practice leaders, clinicians, and operations staff to implement appropriate evidence-based practices into routine clinical care. They work within distinct disease- or condition-specific QUERI Centers and utilize a standard six-step process:

1) Identify high-risk/high-volume diseases or problems.
2) Identify best practices.
3) Define existing practice patterns and outcomes across the VA and current variation from best practices.
4) Identify and implement interventions to promote best practices.
5) Document that best practices improve outcomes.
6) Document that outcomes are associated with improved health-related quality of life.

Within Step 4, QUERI implementation efforts generally follow a sequence of four phases to enable the refinement and spread of effective and sustainable implementation programs across multiple VA medical centers and clinics. The phases include:

1) Single site pilot,
2) Small scale, multi-site implementation trial,
3) Large scale, multi-region implementation trial, and
4) System-wide rollout.

Researchers employ additional QUERI frameworks and tools, as highlighted in this *Series*, to enhance achievement of each project's quality improvement and implementation science goals.

The specific QUERI Centers are the operational arm of QUERI. Their establishment is determined by VA's Health Services Research and Development Service (HSR&D), in conjunction with clinical management. The existence of the HIV/Hepatitis QUERI Center, in part, reflects the fact that the VA is the largest single provider of HIV care in the United States, providing care for more than 20,000 veterans with HIV, or roughly 40% of the nation's HIV-infected veteran population [1,7]. The original mission of the HIV/Hepatitis QUERI Center was to increase access to, and uptake of, evidence-based HIV care, and to improve the care that veterans receive for their disease and associated comorbid diseases. More recently, the scope of this Center has expanded to include veterans with chronic viral hepatitis.

Previous accomplishments of the HIV/Hepatitis QUERI Center include the development of effective decision support tools for HIV providers; analyses of the cardiovascular and cerebrovascular risk from long-term, highly-active antiretroviral therapy; determination of the scientific rational for early identification of HIV infection; development and assessment of strategies to improve medication adherence; and validation of the utility of rapid tests for diagnosis of HIV infection in the VA [8-13].

The purpose of this paper is to provide an illustrative case study that demonstrates work done by the HIV/Hepatitis QUERI Center, and to apply the QUERI 6-step/4-phase framework to the development and implementation of a program to improve rates of HIV diagnostic testing in VA medical care facilities.

The key issues addressed in this paper focus on how the QUERI framework guided our efforts to: 1) identify specific gaps in care, barriers to diagnosis, and the loci within the VA where interventions to improve HIV diagnostic testing would have the greatest impact; 2) develop an intervention to improve diagnostic testing; and 3) implement a staged-series of studies to evaluate the effectiveness of this program. We also discuss how we used known quality improvement strategies to change the behavior of primary care providers in regard to HIV testing.

In the following sections, we show how Steps 1–3 of the QUERI process were followed to demonstrate that undiagnosed HIV infection is a high-risk, high-volume clinical issue (QUERI step 1), that there are evidence-based recommendations for HIV testing (QUERI step 2), and that there are gaps in the performance of HIV testing within the VA healthcare system (QUERI step 3).

Based on this foundation, and with the support of theoretical and empirical considerations, we have developed and initiated a two-site (station) pilot project (QUERI step 4/phase 1) with the goal of refining our interventions to improve rates of HIV testing prior to launching a small-scale, multi-site evaluation in 5 separate stations (QUERI step 4/phase 2).

## Methods

### Step 1: Priority Condition/Issue: Is undiagnosed HIV infection a high-risk, high-volume clinical issue within the VA?

The observation by the CDC that 25% of HIV-infected patients in the United States do not know their status is of great relevance to the VA [1] because the VA treats more than 20,000 veterans with HIV per year [1]. If, as in the rest of the country, 25% of HIV-infected VA patients do not know their status, approximately 7,000 veterans are at risk of being diagnosed with HIV and treated only when they become symptomatic and severely immunosuppressed. Thus, since HIV patients benefit greatly from early diagnosis and treatment [2,3], increased HIV testing in the VA can substantially reduce the number of newly diagnosed veterans who present with concurrent complications of profound immunodeficiency [3,14] and extend survival for the average HIV-infected patient by 1.5 years [2,3].

The decision by the HIV/Hepatitis QUERI Center to focus on HIV testing as a priority within HIV quality improvement was based on the above observations. This decision was approved by the nationally constituted executive committee of the HIV/Hepatitis QUERI Center. QUERI processes require that this prioritization needs to be re-justified on an annual basis through the development of a strategic plan that is reviewed and approved by VA/HSR&D Central Office appointees, representing both researchers and clinical management.

### Step 2: Evidence-based Practices: Are there evidence-based recommendations for HIV testing?

The U.S. Preventive Services Task Force (USPSTF) gives a Grade A recommendation to HIV screening for all adolescents and adults who are at increased risk for HIV infection or who receive health care in a high-prevalence setting, such as where ≥ 1% of the patient population is known to be HIV-infected (Table 2) [15]. The VA has endorsed the USPSTF guidelines for HIV infection and has identified testing for HIV as being a high priority [16].

**Table 2:** US Preventive Services Task Force Grade A Recommendations for HIV Screening*

***Clinicians should screen for HIV all adolescents and adults at increased risk for HIV***.
A person is considered at increased risk for HIV infection if he or she reports one or more individual risk factors or receives health care in a high-prevalence or high-risk clinical setting. HIV Risk factors include:
• Men who have had sex with men after 1975.
• Men and women having unprotected sex with multiple partners.
• Past or present injection drug users.
• Men and women who exchange sex for money or drugs or have sex partners who do.
• Individuals whose past or present sex partners were HIV-infected, bisexual, or injection drug users.
• Persons being treated for sexually transmitted diseases (STDs).
• Persons with a history of blood transfusion between 1978 and 1985.
• Persons who request an HIV test despite reporting no individual risk factors.

High-risk clinical settings:
• STD clinics,
• Correctional facilities,
• Homeless shelters,
• Tuberculosis clinics,
• Clinics serving men who have sex with men, and
• Adolescent health clinics with a high prevalence of STDs.

High-prevalence settings:
• High-prevalence settings are defined by the CDC as those known to have a 1% or greater prevalence of infection among the patient population being served.

***Clinicians should screen all pregnant women for HIV***
*As defined by the US Preventive Services Task Force, screening means counseling and testing.

Adapted from [15]

The cost-effectiveness of HIV testing in the VA is well established. As shown by a HIV/Hepatitis QUERI-affiliated investigator and others, for a population with a prevalence of HIV infection of ≥ 1%, the cost of one-time screening for HIV infection is $15,078 per quality-adjusted life-year gained [2]. Analyses that consider the relationship of diagnosis and treatment on HIV transmission show that the cost of routine HIV screening is < $50,000 per quality-adjusted life-year gained, unless the prevalence of HIV infection is <0.05% [2]. This level of cost effectiveness, which matches that of many well-accepted procedures such as performing colonoscopy for colorectal screening [17], provides a strong argument in favor of implementing HIV testing programs, especially as VA patients have rates of HIV much higher than the 0.05% lower bound of cost-effectiveness [18].

### Step 3: Quality/Performance Gaps: Are there gaps in the performance of HIV testing in the VA?

Previous studies done by HIV/Hepatitis QUERI, the VA Public Health Strategic Healthcare Group (PHSHG, a dedicated national VA program office for guiding HIV care services), and other VA groups have shown that only 30 – 50% of VA patients with known, documented risk factors have undergone HIV testing [11,16]. Furthermore, at the time of HIV diagnosis half of the veterans have advanced levels of immune suppression. These veterans have, on average, 3.7 years of VA care before their HIV is diagnosed [19].

To confirm and extend these data, we evaluated the rates of HIV testing in veterans seen in the five southern California and Nevada VA facilities (otherwise referred to as Veterans Integrated Service Network 22 or VISN 22). We found that between January 1999 and December 2004 only 30% of the 45,776 at-risk veterans (i.e., VA patients with positive laboratory tests or diagnostic codes for hepatitis, sexually transmitted diseases, and/or substance abuse) had been tested for HIV infection. The rate of testing for at-risk patients ranged from a low of 8% in primary care clinics to a high of 47% in substance abuse clinics. The low testing rates and large number of at-risk veterans in VA primary care clinics (nearly ten times as great as in substance use clinics due to the vast differences in the size of these clinic populations) pointed to the need for an intervention to focus on improvements in HIV testing performance in VA primary care clinics. To better understand the source of gaps in care and to discern facilitators that would improve current practice, we reviewed VA policies regarding HIV testing and surveyed providers' practices and attitudes regarding HIV testing at two VA facilities. Following, we present these findings.

#### Organizational factors

Public Law 100–322 requires that VA patients provide voluntary informed consent for HIV testing and that providers document pre- and post-test counseling [16]. In addition, many VA providers regard HIV testing and pre- and post-counseling to be the sole provenance of specially trained HIV counselors [20]. Furthermore, standard policy has been to require all patients with positive or negative test results to come back to clinic for face-to-face, post-test counseling. The post-test counseling appointment is problematic, as many VA providers do not have sufficient appointment slots to allow for timely in-person patient notification of test results (i.e., within 1–2 weeks of the test).

#### Provider willingness and ability to perform HIV testing

Surveys of a convenience sample of 30 VA primary care providers indicated that lack of knowledge of individuals' risk factors for HIV infection, the time requirements to fulfill necessary counseling processes [20,21], and anxiety about post-test counseling patients who have positive test results [14,22] were substantive barriers to ordering HIV tests.

#### Patient acceptance of HIV testing

A systematic review of 62 studies found that acceptance rates of voluntary HIV testing in the United Sates varies from 11% to 91% [23]. Importantly, this review found that higher acceptance rates were associated with confidentiality protections (strongly upheld by VA policy and procedures), as well as the provider's belief that testing would be beneficial.

### Step 4: Identify and implement interventions to promote best practices

Here we describe our QUERI step 4 activities in further detail. We are now nearing the end of phase 1 of step 4, wherein we are conducting a two-station (two-site) pilot project.

### Collaborating with clinical services to design an appropriate intervention

We explicitly sought broad institutional support for this project. This included the support of the VA PHSHG, and the VISN 22 Director, Quality Improvement Council, Clinical Practices Council, and the Clinical Performance Committee. VISN 22 leadership agreed to make HIV testing a performance monitor, to support installation of the HIV Testing Clinical Reminder, and to participate as full partners in enhancing station accountability. After obtaining national and regional support, we also made presentations to, and received support from the Medical Executive Committees, Chiefs of Staff, Ambulatory Care Leadership, primary care teams, and the HIV Coordinators at our two intervention stations. Receipt of all this support was greatly facilitated by the products generated from QUERI steps 1–3.

### Development and initiation of a program to improve rates of HIV testing

#### Conceptual basis of the implementation interventions

As required by the QUERI process, we paid careful attention to the selection of a quality improvement model upon which to base our intervention program. We elected to base our program upon the *Chronic Care Model *(CCM). This decision was based on the previous, wide success of interventions based on the CCM precepts to improve clinical preventive care services [24,25]. Further guidance for this implementation strategy was provided by Rogers, whose germinal work on diffusion promotes the use of opinion leaders or champions to facilitate change or innovation adoption, and highlights the importance of individuals' social networks, organizational leadership and structure [26].

Key components of the CCM include a clear definition of optimal care and enumeration of targeted patients, i.e., offering HIV-testing to at-risk patients; a road map for changing the system; and an effective improvement strategy [27]. Effective CCM implementation strategies also contain the following elements: decision support, clinical information systems, delivery system design, and patient self management [24,28-30]. Therefore, we developed an implementation strategy that uses: clinical reminders to provide *decision support*, audit/feedback as a *clinical information system*, and organizational change to achieve an appropriate *delivery system design*. These activities are complemented by academic-detailing and social marketing interventions to achieve *provider activation *to ensure that providers have the skills and motivation to improve their performance [24,29-31]. Both the CCM and the Institute for Healthcare Improvements Breakthrough Series have identified the need for provider activities to transform and sustain changes in group norms [24,29,30]. Thus, we chose to implement a multi-faceted provider activation program that includes *academic detailing *and *social marketing *[25]. Finally, we promoted HIV counseling to increase *patient self-management *by wide scale publicity of the HIV testing program in clinic waiting rooms and check-in areas.

Table 3 summarizes the relationships between the barriers to HIV testing, the content of the planned implementation program, and the relationship of each intervention to the elements of our CCM-based implementation strategy, which was augmented by academic-detailing and social-marketing interventions (provider activation). In the following section, we discuss the components of these interventions and the methods of delivery in more detail.

**Table 3:** Relationship between Identified Barriers, Elements of the Chronic Care Model and Implementation Strategy

**Barrier to HIV testing**	**Content of implementation intervention**	Relationship to Chronic Care Model*
Lack of knowledge of HIV risk factors.	HIV Testing Clinical Reminder	Decision support
VA providers regard HIV testing and counseling work to be the responsibility of specially trained HIV counselors.	Audit/feedback reports Training to use revised VA HIV Consent Form Academic detailing, social marketing	Clinical information system Delivery system design Provider activation
Lack time required to fulfill counseling processes.	Provider training regarding streamlined counseling Nurse-based pre-test counseling Academic detailing, social marketing	Delivery system design Provider activation
Face-to-face post-test counseling is difficult to arrange within two weeks of the test.	Telephonic notification of negative test results. Academic detailing, social marketing	Delivery system design Provider activation
Anxiety about post-test counseling for positive test results.	Assistance provided by HIV counseling services. Academic detailing, social marketing	Delivery system design Provider activation

• Provider activation is not, strictly speaking, a component of the CCM based implementation strategy, but was rather employed to augment the effectiveness of the other interventions [24;29;30].

### Components of the implementation program

#### Decision Support

**Figure 1:**
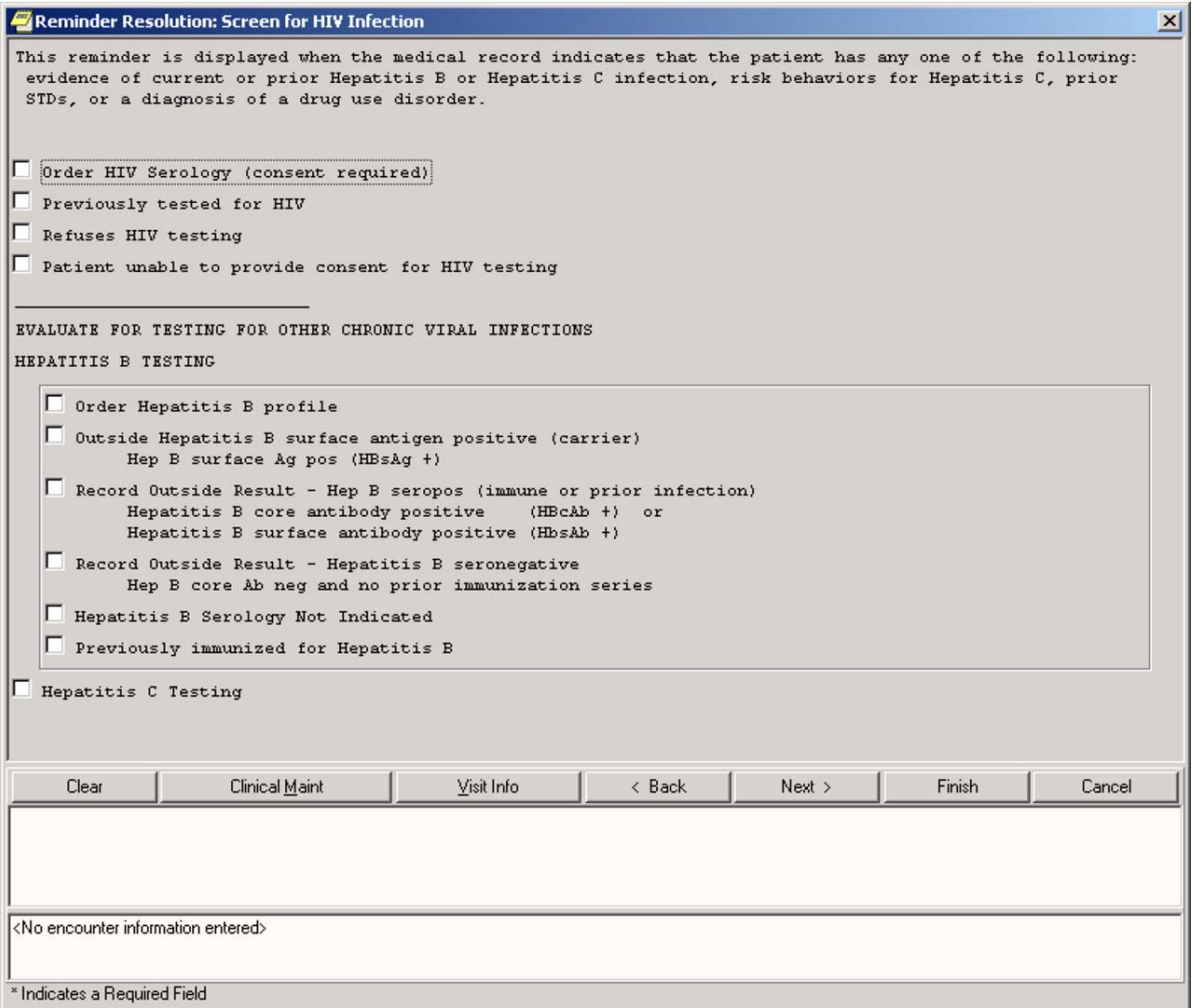
The HIV Testing Clinical Reminder is activated whenever a patient with HIV risk factors, who has not been previously tested for HIV, has an encounter with a healthcare provider. The format of the Reminder is as pictured.

To leverage institutional resources, we implemented a real-time, electronic clinical reminder that had been developed by the VA PHSHG to identify veterans at higher than average risk for HIV infection – and to encourage providers to offer HIV testing to such individuals (Figure 1). Widely used to implement quality improvement, clinical reminders are well-suited for use in the VA because of the system-wide computerized patient record. The HIV Testing Clinical Reminder is triggered by prior evidence of infection by Hepatitis B or C infection, illicit drug use, sexually transmitted diseases (STDs), homelessness, and/or documented risk factors for Hepatitis C infection. All these data elements can be automatically extracted from the VA electronic medical record. The reminder is resolved (i.e., appropriately addressed) by: ordering an HIV test, recording the result of an HIV test performed elsewhere, indicating that the patient is not competent to consent to testing, or specifying that the patient refuses HIV testing. Once resolved, the reminder is no longer triggered.

The use of clinical reminders in individual patients, when combined with audit/feedback and organizational changes, has been shown to improve performance of vaccination, cardiovascular risk reduction, and breast and colorectal cancer screenings [9,10,28,31-33]. Electronic clinical reminders are a standard well-developed technology with which VA providers have great familiarity and have been shown to be well suited to improve performance of tasks similar in nature to HIV test ordering and counseling. Furthermore, our previous work has shown that use of clinical reminders contributes to 10–30% increases in the rates of appropriate clinical interventions in VA HIV-infected patients [9,10]. Thus, this implementation intervention satisfies the FITT (fit between individuals, task and technology) framework for assessing the suitability of using this intervention in our strategy [34].

#### Clinical information system

**Figure 2:**
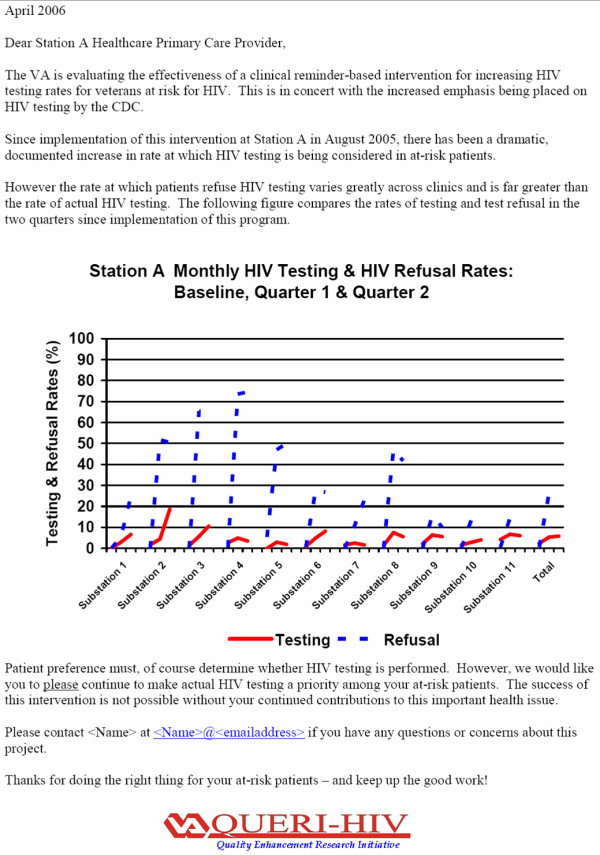
Sample Audit/Feedback letter. The original report identifies the station and sub-stations by name rather than as "Station A" and sub-stations 1–11. The data provided in this letter indicate the number of persons with identified HIV risk factors who were tested for HIV or who were indicated to have refused HIV testing. No patient had previously been tested for HIV. These results, which represent incident HIV testing and refusal rates, differ from the data given in the results section that indicates the cumulative proportion of patients with identified HIV risk factors who had ever been tested. Achievement of 100% on the Y axis would indicate that all at-risk patients were offered HIV testing as denoted by records indicating that HIV testing was performed or refused by the patient.

We designed an audit-feedback system, wherein healthcare providers are informed of group performance in regard to HIV screening rates in at-risk patients. A meta-analysis of 85 trials demonstrates that the use of audit-feedback is effective in improving practice, especially when baseline adherence is low [35]. We have distributed audit-feedback reports (Figure 2) to senior medical center-level clinical managers, outpatient clinic managers, and primary care team leaders at the Los Angeles and the San Diego VA stations. The contents of the reports have been discussed during academic detailing visits to primary care team meetings, and in the social marketing campaign. Informal provider feedback regarding the content of these reports has been positive.

#### Provider activation

We implemented a provider activation program that includes academic detailing and social marketing [25,35,36]. This approach recognizes that the engagement of providers and the use of multiple modalities are necessary to achieve and sustain the transformation of group norms and maximize quality improvement [24,25,29,30,35]. The goal of these activities was to engage providers and influence their attitudes, skills and habits regarding offering HIV testing to at-risk patients. We also used these interactions to reinforce more formal educational efforts. Particular attention was paid to: increasing providers' sense of responsibility for ordering HIV tests, emphasizing the use of streamlined pre-test counseling, reinforcing the use of telephonic notification of negative test results, and assuring providers of the availability of assistance for notification of persons with positive test results.

The academic detailing component of the project involves multiple presentations by clinical champions (physician and nursing staff), supplemented by project staff to the primary care team meetings and educational sessions. We have specifically targeted primary care clinic leaders as local, organizational opinion leaders [25]. We used social marketing with providers to reinforce the importance of changing their practice regarding HIV testing, and further motivate them to do so. Social marketing entails the development of a shared buy-in to the overall goal of the behavior change and is predicated on *social exchange theory*, which borrows from social and behavioral science doctrines in emphasizing the client/patient/provider as the starting point [37]. The social marketing element includes regular informal discussions of the basis for and benefits of increased rates of HIV testing by project staff during frequent ad hoc visits to the primary care clinic and presentations to sub-station and clinic leadership.

As per the precepts of social marketing theory [37], we have undertaken: *audience segmentation *for focused detailing to nurses, mid-level providers and physicians; *channel analysis *to optimize the setting and materials for these audience segments; *goal orientation *to keep stakeholders focused on why they are involved (i.e., emphasizing the differing tasks by nurses [pre-test counseling ] and physicians [order entry]); and *process tracking *to monitor progress and provide feedback for refinement and revision of strategies (i.e., through audit-feedback and through formative evaluation), which can be considered an intervention [38].

Based on survey responses by physicians, mid-level providers, nurses, and case managers, we also have developed provider education materials to supplement the face-to-face training conducted by project staff that focus on preparing providers to use the reminders effectively, making providers aware of HIV risk factors not captured by the reminder (i.e., multiple unprotected sexual contacts), and increasing provider comfort and abilities to provide pre- and post-test HIV counseling.

In our step 4/phase 1 project, all social marketing has been performed by senior project staff. Using the insights gained in the two-station pilot project, as we progress down the pipeline to a step 4/phase 2 small-scale, multi-station evaluation we will rely on a train-the-trainer model to activate local champions. Project staff will support the local champions with regular visits (monthly for the first 3 months of local implementation, and then quarterly), weekly telephone conference calls and e-mail support.

#### Delivery System Changes

We have strived to ensure that all providers are trained to use a recent revision of the VA HIV Consent Form [39]. This new document includes all the necessary elements of pre-test HIV counseling, and thus facilitates the consent process for healthcare providers who lack specific training regarding the performance of HIV counseling. We also have encouraged nurse-based rather than physician-based pre-test counseling [40]. Nurses perform many educational, health promotion, and disease prevention tasks as well as physicians [41]. Organizational changes that shift responsibilities from physicians to other personnel, most often nurses, are effective in improving preventive care. Moreover, we have encouraged providers to use a streamlined HIV counseling process that covers all the required elements of HIV pre-test counseling and reduces the time of pre-test counseling to 2–3 instead of 10–15 minutes, with some counseling lasting as long as 35 minutes [42]. Similarly, we have reduced the logistical challenges of post-test HIV counseling [16]. Given the gravity of the information, post-test counseling for persons with new positive HIV test results strongly warrants face-to-face counseling. In contrast, we have alerted providers that for patients with a negative result, post-test counseling can be very brief and can be done via the telephone [43]. To ensure compliance with post-test counseling requirements, we have distributed sample scripts for transmitting the results of the test.

In addition to being theory-informed, these interventions are informed by empirical evidence provided by studies in urgent care clinics, emergency departments, and STD (sexually transmitted disease) clinics that show increased testing rates and patient receipt of test results after implementation of structural changes, such as improved staff training in pre- and post-testing screening, introduction of streamlined counseling, and substitution of telephonic post-test counseling in place of a required return visit for face-to-face notification [43].

### Implementation

The intervention program is being put in place for one year in the primary care clinics of stations A and B in VISN 22. The three remaining stations in VISN 22 (stations C, D and E) served as controls. These facilities each provide care to 37,000 – 80,000 veterans per year. Facilities were assigned to the active or control arms by convenience. All facilities, except for one of the controls, consisted of an inpatient center plus one or more geographically dispersed sub-stations in which primary care and specialty services, including mental health and substance abuse treatment programs, were provided by academic staff physicians, post-graduate medical trainees, and mid-level providers. In addition, these facilities also provided primary care in other sub-stations staffed solely by non-academic physicians and mid-level providers. At the remaining control facility, care was provided only in outpatient sub-stations by providers who generally did not have an academic affiliation.

The decision support aspects of the intervention and policies regarding performance of HIV consenting and counseling have been implemented at all sub-stations and all clinics at the active facilities. However, the provider activation component of the program (i.e., academic-detailing and social marketing) has been fully implemented only in the primary care clinics at the two largest sub-stations at Facility A (out of a total of 11 sub-stations) and at the two largest sub-stations at Facility B (out of a total of 6 sub-stations); these sub-stations account for 46% and 69% of patients seen in primary care clinics at Facilities A and B, respectively. The other, smaller and geographically distant sub-stations differ in that e-mail and telephonic outreach largely replace personal outreach to promote academic detailing; all other tools (i.e., audit/feedback, provision of printed materials such as e-mail communications, pocket cards, posters and flyers, and removal of organizational barriers) are the same at all stations. The audit feedback program is directed at all providers in every primary care clinic.

### Evaluation plan

The primary endpoints of this step 4/phase 1 two-station pilot project are the effect of the implementation program on the rates of resolution of the HIV clinical reminder and of HIV testing in patients with identified HIV risk factors. A multi-level, logistic regression analysis of the HIV testing rate will be done to adjust for the covariates at patient, provider, and sub-station levels – and for clustering.

We have obtained information regarding all inpatient and outpatient patient encounters within VISN 22 from a pre-established network database. For patients seen in outpatient clinics before and/or after the intervention, we obtained relevant laboratory tests, diagnosis codes, and health factors to determine if they were at increased risk for HIV.

Data regarding non-VA HIV testing, refusal of HIV testing, and incompetence for HIV testing were extracted from the standardized VA clinical reminder software package. Patients were defined as having been tested for HIV if there was documentation of HIV testing done within the VA healthcare system. Veterans were defined as having been evaluated for HIV if there was electronic documentation of prior HIV testing within the VA or elsewhere, patient's refusal to be tested, or patient's incompetence to consent for testing. Information regarding prior HIV testing within the VA was obtained through the VA electronic laboratory records, whereas information regarding outside testing, test refusal, and incompetence to consent was collected through responses to the HIV testing clinical reminder.

In addition to the data abstracted from the VISN 22 database, we also obtained a list of primary care providers classified into provider types (senior staff physicians, mid-level providers, physician assistants, and post-graduate medical trainees) from the primary care administration staff at Facilities A and B. The data on provider types were used to compare HIV testing and evaluation performance across different types of primary care providers.

As previously noted, we fully implemented the provider activation module only at the largest sub-stations at the intervention stations. This design allows us to assess whether this module, which is the most labor intensive component of our implementation strategy, independently contributes to improvement in the rates of HIV screening and testing. In addition, we are conducting formative evaluations to further refine our program and assess the organizational factors that determine the generalizability [38]. The overall aim is to better understand the influences that have an impact on the success of the implementation program by identifying contextually relevant factors (i.e., facilitators and barriers) and assessing the degree to which behaviors that led to improved testing performance become part of routine practice [38]. Semi-structured interviews with key informants will provide qualitative data regarding the effectiveness of the mode used for providing audit/feedback, the usefulness and usability of the testing reminder, and the efficiency of the consenting/counseling process. Interview questions will employ rapid ethnographic assessment methods to explore the ecological context of HIV testing [44].

To assess the degree to which behaviors leading to improved testing performance are institutionalized (i.e., become embedded in standard operating procedures), leading clinicians and administrators at each sub-station will complete a Level of Institutionalization survey. The instrument measures four sub-systems that support routine use of an innovation: *production*, where it must be integrated with other routine clinical services; *maintenance*, where employees must support it; *supportive*, where it must have a stable source of funding; and *managerial*, where it must be assigned to a specific service, staff must have written job descriptions, and performance is required to be measured and reported [45].

Finally, we are generating a comprehensive analysis of the workload and implementation costs of HIV screening and testing programs, using Business Case Modeling, a method for constructing data-driven models that forecast costs under varying specified conditions that support managerial or technical decision-making. This is warranted as the models of the cost-effectiveness of HIV screening do not address the upfront costs of implementing screening programs across differing clinic settings [2,3,46].

## Results

Preliminary unadjusted data show an increase in the cumulative rate of reminder resolution (i.e., rate at which the reminder was appropriate addressed) from 22% to 64% at Station A and from 33% to 70% at Station B during the first seven months of this project. Although the amount of salutary change varied across the geographically dispersed sub-stations of the two study stations, all sub-stations showed substantial increases in the rate of resolution of the HIV Testing Clinical Reminder. In contrast, no change in the reminder resolution rate was seen at the other VISN 22 stations where the intervention was not undertaken. Actual monthly rates of HIV testing (as opposed to other means of resolving the HIV Testing Clinical Reminder) increased 3–5 fold. In contrast, no change in HIV testing rates was seen in the control stations. As compared to at-risk patients seen at the control facilities, at-risk patients at the intervention facilities were more likely to be African American or to have a history of either illicit drug use or homelessness. Otherwise there were no significant differences among the demographics and risk factors for HIV risk factors of veterans who received care at the intervention versus the control facilities.

Of note, we found an unexpected high variation in the ratio of HIV test refusals to test performances with some sub-stations recording 10–20 times as many refusals as actual tests, whereas other sites had fewer refusals than tests. These results are at odds with studies that show higher rates of acceptance of HIV testing [23]. Our preliminary analyses suggest that some providers take more care than others in terms of informing patients of the benefits of knowing their HIV status. Furthermore, the rates of resolving the HIV clinical reminder and the ratio of HIV test refusal to performance appear to differ substantially among provider types (i.e., senior staff physicians vs. mid-level practitioner vs. post-graduate medical trainee), with trainees performing far less well than other providers.

Thus far, our preliminary formative evaluation also indicates that further work needs to be done to address station-specific barriers to HIV testing. Particular areas of concern include the development of procedures to address variances in the adoption of new technologies, such as the use of new, electronic paperless versions of the VA HIV consent form. Other important station-to-station differences involve workflow patterns. For example, station B routinely utilizes more nursing personnel for normal clinic routines. Therefore, their intake nurses were expected to share consenting and counseling responsibilities to promote efficient processing of at-risk patients. The process of addressing reminders at station A, on the other hand, is more physician-driven and requires targeting activation strategies to their different role and skill set. Our step 4/phase 1 two-station project also has allowed us to identify aspects of the HIV testing clinical reminder that lead to provider dissatisfaction. In particular, providers would like the reminder to clearly indicate what specific factor(s) triggered the HIV Testing Clinical Reminder, so that they can better counsel patients as to why HIV testing is relevant to the veteran's circumstances. Providers also have expressed a strong belief that homelessness, *per se*, is not a substantial risk factor for HIV infection, and that HIV testing may not be relevant to persons with a limited life expectancy. Finally, providers perceive the layout of the reminder to be unduly complicated.

## Discussion

This project has greatly benefited from practical guidance and consistent support of the VA QUERI program. For example, a step 3 study by Owens et al., showing that fewer than half of at-risk VA patients were tested for HIV, formed an important part of the basis of our rationale to proceed to step 4 [11]. These results were confirmed by further evaluations supported by core funding provided through the QUERI program. Moreover, the QUERI process guided our analysis of the importance of improving VA HIV testing rates, our assessment of the gaps in care, and the development of a multi-faceted project with broad-based institutional support to close this quality gap. Although the full analysis of our step 4/phase 1 two-station pilot project results is pending, and efforts to gauge the sustainability of our project are only just beginning, release of our preliminary results [47] has attracted considerable attention from other VA facilities, some of which are undertaking similar efforts to implement the electronic HIV Testing Clinical Reminder. The results of such implementations, which are being done without formal provider activation components, are not yet available. Specific issues of particular importance to our implementation program are discussed below.

### Importance of leadership and team buy-in

The successful implementation of this project and the favorable preliminary results could not have been achieved without support at multiple administrative and clinical levels. The VA PHSHG provided invaluable support by promoting HIV testing within the VA, and by developing the HIV Testing Clinical Reminder. Their backing facilitated our ability to get the support of VISN 22 network leadership. In turn, this enabled us to elicit support for the institutionalization of the reminder, organizational changes, and implementation of the provider activation program at the individual stations where the step 4/phase 1 two-station pilot project was launched, and, subsequently to get the buy-in of individual primary team leaders. Ultimately, provider support is crucial. Acquiring this support required us to address issues of concern to providers, most notably facility-specific barriers to HIV testing, variances in the prevalence of HIV risk factors, issues related to the use of the reminder, and factors that contribute to variances in actual HIV testing versus refusal of HIV testing.

### Station-specific barriers

Despite being a single healthcare system, there are substantial variances in local policies, processes, and institutional culture within the VA that must be addressed by station-specific customization of implementation projects. For example, differences between the processes of HIV consenting and counseling in stations A and B have required separate activation/education sessions with nursing staff regarding mandated requirements of the counseling/consenting process and the legal boundaries of their scope of practice. We also have found that further variations in training needs arise from differences in the mixtures of providers, such as the degree to which patient care is delivered by mid-level providers, post-graduate medical trainees, part-time physicians, contract employees, and full-time staff physicians. Furthermore, one station only allowed trained HIV counselors, who were positioned in key clinics and sub-stations, to order HIV tests or to perform pre- or post-test counseling. We needed to work with key stakeholders to change these policies. In addition, the rate of implementation of new technology varies. Thus, while electronic consent for HIV testing is available [48], acceptance of paperless consent requires concomitant local policy changes. Similar issues pertain to the implementation of rapid-testing for HIV infection – a procedure that has excellent patient acceptance in the VA [12].

### Variations in the prevalence of HIV risk factors

Although individual providers generally have a panel size that is normalized to the time they spend in clinic, the proportion of veterans with HIV risk factors can vary dramatically. For example, a much higher proportion of patients trigger the HIV Testing Clinical Reminder in clinics that care for large numbers of veterans with drug use histories or hepatitis C infection. Providers in such clinics warrant attention to ensure that they do not feel overwhelmed by the burden of work precipitated by responding to these reminders.

### Issues related to use of the reminder

As per the FITT model, reminders require customization to the needs of providers [34]. We have found that providers benefit from having ready access to the specific factor(s) that trigger the HIV Testing Clinical Reminder for an individual patient. In addition, it is important that the factors that trigger the Reminder be as closely linked to actual risks of HIV infection as possible. In this regard, we have found that the triggers for a diagnosis of homelessness are sufficiently non-specific (i.e., loss of housing for a few days) as not to warrant inclusion in the reminder. Similarly, the process triggered by the Reminder needs to be of potential benefit to the patient. Thus exclusion of persons with a very limited life expectancy is appropriate. Based on these findings, we are adopting a revision of the Reminder that addresses these issues.

### HIV Test ordering versus HIV test refusal

The substation-specific variances in the ratio of HIV test orders to HIV test refusal was an unexpected finding. In response we are seeking to better understand the determinants of these behaviors. We anticipate that these will include provider comfort with HIV testing and site-specific barriers to HIV testing. We will also determine whether particular demographic factors, HIV risk factors or physician factors, are associated with patient refusal of HIV testing. Based on our findings, we will design a broader campaign of provider activation, such as reminding providers of patient acceptance of HIV testing [23] and delivery system changes to alleviate this discrepancy.

In summary, the strengths of our study include a quasi-experimental design that permits comparison of the effect of the implementation program on changes on HIV testing and evaluation rates – before and after the program is active and with control stations. This program relies heavily on the built-in quality improvement infrastructure in the VA, including the electronic medical record, clinical reminder software, and familiarity with performance measurements. Although unique aspects of the VA infrastructure will make it more difficult to generalize introduction of our process to healthcare systems that do not have these tools, such systems are becoming increasing common. Other limitations of our current findings include our incomplete qualitative process (or formative) evaluation to better understand the influences that impact the success of the program. In ongoing work we hope to identify contextually relevant factors (i.e., facilitators and barriers) and assess the degree that behaviors leading to improved testing performance become part of routine practice [38]. Areas of particular interest will be evaluating the contribution of nurse-based vs. physician-based HIV testing and evaluation, as well as the role of intensive provider activation, as this is the most costly and time consuming activity. Finally, although the overall cost-effectiveness of HIV screening is not in doubt, these models do not address variations in the complexity of screening programs across differing clinic settings, nor the costs of implementing a screening program [2,3,46].

### Next steps

After completing our 12-month QUERI step 4/phase 1, two-station (site) pilot project, we will extend our work by performing a QUERI step 4/phase 2, small-scale, multi-site evaluation at all five VISN 22 stations. This will allow us to conduct a formal, multi-site formative and analytical evaluation, determine the sustainability of our project in our initial two-stations, determine the institutionalization of behaviors by our two-station pilot, and further refine and evaluate our implementation program.

If the phase 2 results are favorable, we plan to study the refined program in multiple geographic regions (QUERI step 4/phase 3), prior to a national roll-out of the program (QUERI step4/phase 4). We anticipate that with any widespread roll-out there will still be a need for the receiving facility to adapt/contextualize/customize the implementation program to maximize the likelihood of adoption. Finally, QUERI steps 5 and 6 call for documentation that best practices improve outcomes and that outcomes are associated with improved health-related quality of life. Therefore, of ultimate concern is whether increased rates of HIV screening and testing actually increase the rates of early HIV diagnosis, decrease HIV transmission and disease progression, and improve quality of life for HIV-infected and uninfected persons.

## Competing interests

The author(s) declare that they have no competing interests.

## Authors' contributions

MBG drafted the manuscript. CB, TH, HA, TO, AG and SA participated in the design of the study. TH performed the statistical analysis. All authors read and approved the final manuscript.
